# Beyond the Score: Bias Investigations to Improve the Fairness of Board Certification Exams

**DOI:** 10.1002/aet2.70170

**Published:** 2026-04-29

**Authors:** Kevin B. Joldersma, Chadd K. Kraus, Michael Gottlieb

**Affiliations:** ^1^ American Board of Emergency Medicine East Lansing Michigan USA; ^2^ Department of Emergency and Hospital Medicine Lehigh Valley Health Network Allentown Pennsylvania USA; ^3^ Department of Emergency Medicine Rush University Medical Center Chicago Illinois USA

## Abstract

**Objective:**

High‐stakes examinations, such as those used for board certification, must be valid and fair across demographic groups. The American Board of Emergency Medicine (ABEM) developed a structured process for bias and fairness assessment to identify and refine potentially biased examination items.

**Methods:**

ABEM implemented a three‐phase innovation: (1) statistical flagging of potentially biased items using differential item functioning (DIF) analysis; (2) expert panel qualitative review; and (3) holistic content review by the editorial team.

**Results:**

Over an 8‐year period, 3736 items were analyzed. DIF flagged 597 items (16.0%) for review. The expert Bias and Fairness Panel recommended deletion of 62 (10.4% of flagged items) due to construct‐irrelevant bias, most often related to racial bias (53.2% of items recommended for deletion), followed by regional jargon or practice variation (43.5%). The process has been adopted consistently and is being extended to new examination formats.

**Conclusion:**

A structured, theory‐informed bias and fairness assessment process can reduce construct‐irrelevant variance in high‐stakes learner assessments. This can serve as a replicable model for other certifying bodies and medical educators seeking to enhance their approach to assessment.

## 1 Need for Innovation

Ensuring fairness in certification testing is essential to protect the public and maintain trust in emergency medicine board certification. Evidence suggests disparities in scoring on national examinations when analyzed by demographic groups, raising concern about potential bias, even when psychometric validity is otherwise established [1, 2]. Therefore, it is critical to establish a systematic method to identify and address bias in testing items to ensure rigorous assessment of clinical competence and to avoid disadvantaging examinees based on demographics.

## 2 Background

Both Messick's and Kane's validity frameworks highlight fairness as an essential component of construct validity [3, 4]. Two important concepts for evaluating an assessment's fairness are differential impact and bias. Impact is the presence of a difference in pass rates or scores between subpopulations receiving the same assessment [5]. However, impact does not assign a cause or explanation to this differential performance; as such, impact alone is not proof of bias. Impact is, effectively, the mean differences in test scores that occur despite the absence of measurement bias.

Bias, from a psychometric and assessment perspective, is defined as “[t]he systematic under‐ or over‐prediction of criterion performance for people belonging to groups differentiated by characteristics not relevant to the criterion performance” [6]. In the context of emergency physician assessment, this means that an item or assessment is biased if a group performs better or worse than expected for reasons other than the test taker's knowledge or skill in emergency medicine. A biased item might occur if an item writer used jargon to describe a lab or test result that is specific to their workplace, which may be regionally understood but is not nationally used. Contrarily, an item that tests knowledge of sickle cell disease or Lyme disease may have a differential impact but, because this knowledge is what is known as “construct relevant” (i.e., key to the practice of emergency medicine), would not be found to be biased. The determination of whether the source of the impact is relevant to emergency medicine is a complex and holistic process, requiring multiple steps. Given the substantial implications associated with the assessment process by specialty boards, it is important to have a robust and comprehensive process for bias assessment.

## 3 Objective of Innovation

The American Board of Emergency Medicine (ABEM) sought to design and implement a rigorous, scalable process to (1) detect potential bias in individual test items across multiple emergency medicine examinations; (2) remove or revise items demonstrating construct‐irrelevant bias; and (3) create a model for continuous quality improvement that could be exported to other certifying organizations.

## 4 Development Process

ABEM convened a multidisciplinary working group of emergency physicians and psychometricians to design a process grounded in educational measurement theory. Messick's framework provided the conceptual underpinning, emphasizing fairness as integral to validity [3]. This was also informed by a review of the literature and consultation with psychometricians in other organizations [7]. The innovation aligns with constructivist assessment principles, which require that examinations measure intended competencies rather than extraneous factors such as regional terminology.

The ABEM bias investigation process consists of three phases: (1) statistical flagging of potentially biased items using differential item functioning (DIF) analysis; (2) expert panel qualitative review; and (3) holistic content review by the editorial team.

### 4.1 Statistical Flagging Process

ABEM uses a widely accepted statistical analysis and flagging procedure first developed by the Educational Testing Service (ETS) [8]. The broader method, called DIF, consists of identifying a focal group (e.g., women) and comparing it to a reference group (e.g., men). To ensure a fair comparison of the focal and reference groups, examinees completing the assessment are matched on their total test score. Any test taker who does not complete at least 80% of the test is excluded by ABEM's scoring policy. Matched test takers are then compared using the Mantel–Haenszel procedure, which produces a chi‐squared statistic and odds ratio [9]. The recommended minimum sample sizes are at least 200 respondents in both the reference and focal groups [10]. The resulting odds ratio of each item is transformed to the log‐odds scale and then converted to the ETS' delta scale. Positive values indicate the focal group was advantaged, while negative values indicate the reference group was advantaged. The results are effect sizes and are classified by three categories (A, B, or C) according to the magnitude of the DIF [8, 11]. Items in the A category have a negligible or nonsignificant DIF, B items have a small‐to‐moderate DIF, and C items have a moderate‐to‐large DIF. These categories indicate the degree of DIF (i.e., the degree to which one group may be advantaged or disadvantaged by a particular item). Irrespective of the direction of possible favoritism, all items with a C classification are forwarded to the Bias and Fairness Panel.

Importantly, the Mantel–Haenszel chi‐squared, like all statistics, has the potential for both false positive and false negative flagging of items. ABEM uses intentionally broader flagging criteria that can be expected to identify more than the 5% of the items that would be false positive flags due to chance. Although the criteria could be changed to reduce false positives, it would also reduce the number of false negatives, which could allow some potentially biased items to go unscrutinized.

### 4.2 Expert Panel Qualitative Review

The Bias and Fairness Panel is convened twice a year to review the content of items that have been statistically flagged for potential bias. The timing of these panels coincides with the conclusion of a test administration and prior to editorial review to determine subsequent reuse. Each panel is composed of subject and content matter experts who are ABEM‐certified emergency physicians selected via a stratified random selection of volunteers who represent a diversity of ethnicities, gender, and geographic practice locations. Panels generally have 10 to 15 panelists. The panel is moderated by two psychometricians, one of whom is also a trained linguist.

Each panel is conducted first by establishing a common framework and understanding of impact and bias. This is followed by some education in assessment terminology and concepts. The panel is then charged with the responsibility of reviewing all of the statistically flagged items and making a recommendation as to whether an item should be deleted for bias. If an item is classified as showing bias, the panel must determine whether the source of bias is related to an important aspect of emergency medicine. If the source of bias is important or relevant to the field of emergency medicine, the panel is instructed to keep the item. Otherwise, if the source of bias is not important or relevant to emergency medicine, the panel recommends the item be deleted or reworked. Any items that the panel recommends deleting or reworking are forwarded to the ABEM examination editors for holistic review. To err on the conservative side, if any panelist believes an item is biased, it is recommended for deletion.

### 4.3 Holistic Content Review

**FIGURE 1:**
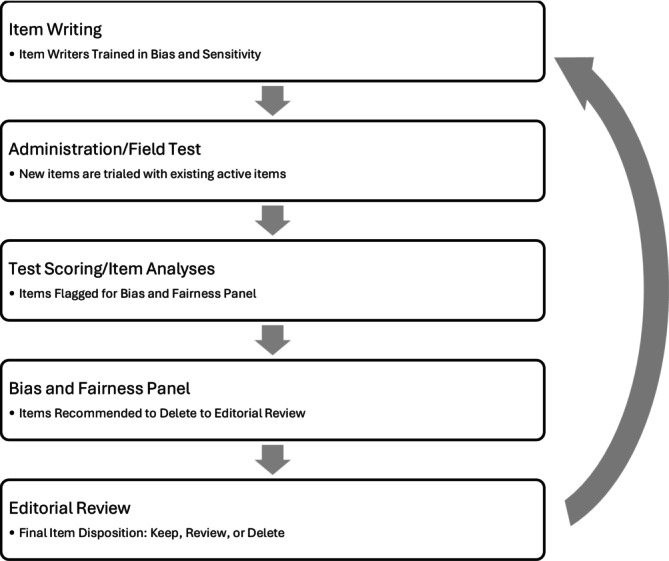
Simplified item development cycle highlighting bias and fairness steps.

The ABEM examination editors review the Panel's recommendations as part of their systematic and holistic review of items. Editors then decide whether to send an item back to the ABEM development group for revision/deletion or if the item should be kept as is. To override the Panel's recommendation, editors must provide a content‐based explanation or argument to keep an item flagged for deletion. In order to have high sensitivity for excluding biased items, reviewers are instructed to have a low threshold for removing an item if there is any question about its potential bias. Items that have been identified as biased by the panel are presented to the editors during the cyclical review of all items that have been administered for the test. During this process, the editors also review the statistical performance of the items, any test‐taker commentary about the items, and psychometric and development staff commentary about the items. Editors must determine, based on all of the available evidence, whether any given item should be kept as is, revised, or deleted (Figure 1).

## 5 Implementation Phase

The bias and fairness process was first deployed by ABEM in 2018 for the in‐training examination (ITE), qualifying examination (QE), and the ConCert examination. Panels met semiannually following test administrations. Demographic data were collected securely from examinees and residency programs to support DIF analysis while maintaining confidentiality.

Over 8 years, 3736 items were analyzed across assessments. Among those, 597 items (16.0%) were flagged for panel review. Sixty‐two items (10.4% of flagged) were recommended for deletion, most commonly due to racial bias (53.2% of items recommended for deletion), followed closely by regionally specific terminology or practice patterns (43.5% of items recommended for deletion) that could disadvantage certain groups. Forty‐eight of these items (77.4%) were ultimately deleted by editors (Table 1).

**TABLE 1:** Bias and fairness process summary.

Test	Year	N analyzed	N flagged	N recommended for deletion	N deleted by editors
In‐training examination	2018	350	30	0	0
	2019	354	34	3	1
	2020	334	37	0	0
	2021	349	31	3	3
	2022	330	38	7	4
	2023	334	24	6	3
	2024	338	39	4	4
	2025	307	9	4	3
Qualifying examination	2018	168	60	3	3
	2019	65	21	0	0
	2020	75	50	2	2
	2021	46	14	2	1
	2022	60	23	8	7
	2023	45	24	4	2
	2024	82	29	7	7
	2025	309	49	4	4
ConCert^a^	2018	110	47	2	2
	2019	39	15	2	2
	2020	32	16	0	0
	2021	9	7	0	0
**Total**		**3736**	**597**	**62**	**48**

^a^ The ConCert examination was discontinued in 2022.

## 6 Outcomes

The bias and fairness process led to the following tangible improvements in examination fairness. First, the process identified and successfully removed approximately 1.3% of the items analyzed due to construct‐irrelevant variance. Second, the results identified process adoption and sustainability, with continued use from 2018 to present across multiple examinations. This was further evidenced by continued deletions occurring through the most recent examinations, demonstrating continued uptake. Third, we have observed a rise in similar models now occurring in other American Board of Medical Specialties' member boards, supporting the innovation's external credibility (*Personal Communication: Addressing Race/Ethnicity Bias on Board Certification Exams. ABMS Board Psychometricians listserv. December 6, 2024*).

## 7 Reflective Discussion

The ABEM bias and fairness process demonstrates that a certifying body can systematically integrate psychometrics, expert review, and item‐writing theory with a goal to enhance fairness without sacrificing rigor. Because the process relies on standard DIF analyses and structured panel review, it can be adapted by other specialty boards, graduate medical education training programs, or even undergraduate program assessments.

For organizations seeking to replicate this model, we identified several key lessons learned. First, it is important to ensure multidisciplinary expertise is utilized throughout the process. Our expert panels included psychometricians, linguists, and a diverse group of emergency medicine physicians to ensure that the statistical findings were interpreted in the appropriate clinical and cultural context. Second, we used conservative flagging thresholds for the DIF to minimize the risk of false negatives. The process began with statistical screening to narrow down the total for expert review, but given the high‐stakes nature of assessment, we emphasized more sensitive thresholds to optimize detection. When seeking to adopt a similar model externally, it would be important to also consider available resources and timing when determining the optimal threshold for other groups. Third, we trained reviewers on the difference between impact and bias to avoid premature conclusions and promote thoughtful deliberation. Fourth, we engaged two separate groups of reviewers to maximize the consideration for each item. This included both the multidisciplinary Bias and Fairness Panel and the examination editor group, with the latter providing an additional holistic lens situated in the context of all available data surrounding the examination. Finally, recognizing challenges with the sustainability of medical education interventions, we initiated measures to optimize their continuation by integrating this into routine item review cycles and utilizing data (e.g., demographics already collected) to minimize additional effort.

There are several limitations that warrant consideration. The Mantel–Haenszel method is sensitive to the sample size and may not capture all forms of differential functioning, particularly with small subgroups. Additionally, demographic data are limited to self‐report from available data; as such, completeness and categorization remain a challenge. Moreover, DIF analyses detect statistical differences but do not establish causality. Therefore, expert judgment remains critical. However, despite diverse, multidisciplinary panels and holistic expert review, the content review remains subjective and can be influenced by the panel selected. Finally, while deletion of biased items improves fairness, it does not directly address structural inequities that may contribute to group performance gaps.

ABEM plans to extend this process to the oral certification examination and other continuous certification assessments as sample sizes allow. The first bias and fairness panel for MyEMCert, ABEM's continuous certification assessment program, was conducted in the fall of 2025. Results were not available for this publication. Future research should explore alternative DIF thresholds and downstream effects to further optimize the process.

## Author Contributions

**Kevin B. Joldersma:** study concept and design, acquisition of the data, analysis and interpretation of the data, drafting of the manuscript, critical revision of the manuscript for important intellectual content, study supervision, take responsibility for the manuscript as a whole. **Chadd K. Kraus:** study concept and design, drafting of the manuscript, critical revision of the manuscript for important intellectual content. **Michael Gottlieb:** study concept and design, analysis and interpretation of the data, drafting of the manuscript, critical revision of the manuscript for important intellectual content, study supervision, take responsibility for the manuscript as a whole.

## Funding

The authors have nothing to report.

## Conflicts of Interest

Drs. Gottlieb and Joldersma are employed by the American Board of Emergency Medicine (ABEM). Dr. Kraus was previously employed by ABEM.

## Data Availability

The data that support the findings of this study are available on request from the corresponding author. The data are not publicly available due to privacy or ethical restrictions.
